# Fully Thermally Decomposable CO_2_-Based Thermoplastic Polyurethane Encapsulation Films for Photovoltaic Cells: Mechanical, Barrier and Recycling Aspects

**DOI:** 10.3390/nano16090503

**Published:** 2026-04-22

**Authors:** Yuting Ouyang, Jizhi Ai, Min Xiao, Dongmei Han, Sheng Huang, Shuanjin Wang, Yuezhong Meng

**Affiliations:** 1 The Key Laboratory of Low-Carbon Chemistry & Energy Conservation of Guangdong Province/State Key Laboratory of Optoelectronic Materials and Technologies, School of Materials Science and Engineering, Sun Yat-sen University, Guangzhou 510275, China; ouyyt7@mail2.sysu.edu.cn (Y.O.); aijzh@mail2.sysu.edu.cn (J.A.); stsxm@mail.sysu.edu.cn (M.X.); huangsh47@mail.sysu.edu.cn (S.H.); 2 School of Chemical Engineering and Technology, Sun Yat-sen University, Guangzhou 510275, China; handongm@mail.sysu.edu.cn; 3 Institute of Chemistry, Henan Academy of Sciences, Zhengzhou 450000, China

**Keywords:** CO_2_-based polyurethane, encapsulants for polycrystalline silicon solar cells, barrier properties, sustainable encapsulants, polycarbonate urethane

## Abstract

The development of sustainable encapsulation materials with tunable thermomechanical properties remains a critical challenge for photovoltaic reliability. Currently, the mainstream encapsulant for polycrystalline silicon solar cells is crosslinked EVA (Ethylene-Vinyl Acetate), which complicates the end-of-life recycling and reuse of modules. There is an urgent need to develop a novel encapsulant that combines excellent barrier properties with thermoplastic recyclability. Herein, we report a novel series of thermally decomposable CO_2_-based thermoplastic polyurethane (PPC-TE) films engineered through the rational design of soft and hard segments. Utilizing polycarbonate diol (PPCDL) and polyether glycol (PEG) as soft segments, we systematically tailor material properties by modulating PEG-to-PPCDL ratios (5–20 wt%) and PEG molecular weights (1000–4000 g/mol). The optimized PPC-TE films exhibit excellent transmittance (>90%), adjustable glass transition temperature (*T*_g_: 35.1 °C~11.6 °C), and remarkable mechanical adaptability (51~92 HA). The PPC-TE films exhibit water vapor permeability (WVP) as low as 14.8 g·mm·m^−2^·day^−1^ and oxygen permeability (OP) of 4.13 cc·mm·m^−2^ day^−1^ at 15 wt% PEG content, surpassing commercial ethylene–vinyl acetate (EVA) encapsulants. Notably, these films demonstrate fully thermal decomposition above 350 °C, facilitating eco-friendly photovoltaic device recycling. Superior adhesion to glass substrates is evidenced by peel strengths up to 37 N/cm (PPC-TE2000-20) and the shrinkage rate is as low as 3%. This work contributes to improving the long-term stability of solar cells and has the potential for large-scale production.

## 1. Introduction

Solar energy has emerged as a cornerstone of the global energy sector over the past two decades [1]. Solar cells, the key component converting solar radiation into electrical energy, have demonstrated remarkable progress [2,3]. Crystalline silicon solar cells dominate the market [4,5], with efficiency steadily improving. In 2022, Longi Green Energy Technology Co., Ltd. achieved 26.81% efficiency using heterojunction technology [6]. Complementing these, thin-film solar cells [7,8,9], including amorphous silicon (a-Si), cadmium telluride (CdTe) [10,11] and copper indium gallium selenide (CIGS) [12,13,14,15], offer cost-effective alternatives. a-Si solar cells provide flexibility, while CdTe and CIGS technologies demonstrate significant commercial viability. a-Si solar cells exhibit stable power conversion efficiency under indoor lighting and are commonly used in smart clothing, electronic skin, and medical patches. CdTe solar cells offer high power output per unit area, making them more suitable for devices that require higher power and must operate in harsh outdoor lighting conditions [10,16]. CIGS solar cells combine high efficiency with lightweight properties, making them an ideal choice for aerospace applications and high-end consumer electronics [13,14]. The adaptability of thin film cells makes them suitable for applications such as wearable electronics and building-integrated photovoltaics (BIPV) [17,18,19,20,21].

Solar cell encapsulation refers to the systematic manufacturing process of tightly bonding fragile photovoltaic cells with components such as high-transmittance glass, back sheets, and frames using encapsulation materials to form a robust, sealed, and directly output DC power unit (i.e., a photovoltaic module). This process is far more than a simple “wrapping”; it is a core technological guarantee that determines whether the module can operate stably in harsh outdoor environments. Solar cells are susceptible to diverse environmental stressors: moisture ingress can cause corrosion and interfacial degradation; oxygen exposure can lead to oxidation; ultraviolet (UV) radiation can initiate photochemical degradation; and thermal cycling or mechanical stress (e.g., from wind or hail) can induce physical damage. Encapsulation serves as a protective barrier, shielding cells from these elements [22] and facilitating optical management to maximize light transmission while minimizing losses [23,24,25]. Encapsulation is a crucial bridge between laboratory cell efficiency and actual power generation revenue in power plants. Without reliable encapsulation, even the highest cell conversion efficiency cannot maintain stable output under the long-term corrosive effects of outdoor wind, rain, ultraviolet radiation, humidity, heat, and thermal cycling. Encapsulation materials directly determine the final output power, long-term reliability, and total lifecycle cost of the module, making it one of the most technologically advanced and value-added core links in the photovoltaic industry chain. A well-designed encapsulation system is essential for achieving a module lifespan of over 25 years.

Current solar cell encapsulants primarily include EVA, polyvinyl butyral (PVB), and polyolefin elastomer (POE). EVA dominates the market due to its excellent optical transparency, adhesion to glass and back sheets, and thermal stability suitable for lamination [26]. However, EVA suffers from significant drawbacks: susceptibility to yellowing under UV exposure and elevated temperatures [27,28,29], which reduces light transmittance and cell efficiency, and relatively high permeability to moisture and oxygen, accelerating long-term degradation. PVB, often used in BIPV for its high impact resistance and glass adhesion [30], requires higher lamination temperatures and exhibits slightly lower optical transmittance than EVA. POE offers good weatherability and processability, but often requires adhesion promoters and may have suboptimal optical properties.

Recent advances highlight thermoplastic polyurethanes (TPUs) as promising high-barrier encapsulants for solar cells owing to their superior optical transparency, tunable thermomechanical properties, and enhanced weatherability [31,32,33,34,35]. Miller David C. et al. compared the durability of polymeric encapsulants, including EVA, PVB and TPUs, for concentrating photovoltaic systems, showing that TPUs exhibit comparable durability for PV encapsulation [36]. For example, fluorinated TPUs achieve high transparency and UV stability while maintaining excellent mechanical properties, including tear resistance and flexibility to accommodate thermal stresses. TPUs also exhibit super weatherability, effectively blocking UV radiation and moisture without significant yellowing. Their thermoplastic nature enables facile processing (e.g., extrusion and lamination) and aligns with the growing emphasis on sustainable manufacturing and recyclability in the solar industry. In 2017, Yang Lijuan et al. [37] reported fluorinated thermoplastic polyurethane materials with fluorinated chain extenders, developing highly transparent fluorinated thermoplastic polyurethane (FTPU) films possessing excellent heat resistance and weather resistance. Subsequently, in 2019, Wang Yilian et al. [38] synthesized fluorine-containing polycarbonate polyurethane (FPCU) films by using hexafluoro bisphenol A (BPAF) as a chain extender. These FPCU films achieved high transparency (greater than 91%). Due to their high transparency, weatherability and thermolability, these TPU films showed high potential application in solar cell encapsulation.

Recently, perovskite solar cells (PSCs) have emerged as promising candidates for next-generation photovoltaics due to their excellent optoelectronic performance, rapidly improving photoconversion efficiency, and compatibility with flexible substrates [39]. Raman R. K. et al. investigated the structure–property relationship of TPU films as an encapsulant for perovskite solar cells. The reported TPUs exhibited robustness, strong adhesion to glass substrates, and adaptability to a low-temperature encapsulation process that avoided the degradation of the perovskite absorber and other organic layers within the device stack [40,41,42]. Yue Y. Q. et al. proposed a transparent TPU film with low moisture permeability to enhance the stability and durability of PSCs. Their work demonstrated compatibility between the TPU encapsulation process and PSC modules, along with the effective prevention of water–oxygen ingress and retardation of efficiency degradation [43].

In this work, we synthesized a novel series of thermal degradable CO_2_-based thermoplastic polyurethane films. Utilizing PPCDL and PEG as tunable soft segments, we systematically investigated the effects of PEG content (5–15%) and molecular weight (1000–4000 g/mol) on key film properties, including optical transparency, thermal mechanical behavior, barrier performance, adhesion, and thermal degradability. The optimized PPC-TE films demonstrate overall performance surpassing EVA, positioning them as a viable, eco-friendly alternative for extending solar cell lifespans and enabling large-scale manufacturability.

## 2. Materials and Methods

### 2.1. Materials

N, N-dimethylformamide (DMF, >99.8%, anhydrous) and tin (II) 2-ethyl hexanoate (catalyst, 95%) were obtained from Aladdin Reagent Co., Ltd. (Shanghai, China). Ethanol (EtOH, anhydrous) was purchased from GHTECH Co., Ltd. (Shantou, Guangdong, China). Polycarbonate diol (PPCDL, OHV = 49.6 mg KOH/g) was acquired from Foshan Zhongtianrong New Materials Technology Co., Ltd., (Foshan, Guangdong, China). 1,4-Butanediol (BDO, 99%) was purchased from MACKLIN Reagent Co. (Shanghai, China). Dicyclohexylmethane 4,4′-diisocyanate (HMDI, ≥90.0%, GC) and polyethylene glycol (PEG, average *M*_n_~1000, 2000 and 4000 g/mol) were also purchased from Aladdin Reagent Co., Ltd. (Shanghai, China).

### 2.2. Synthesis of CO_2_-Based Thermoplastic Polyurethane (PPC-T and PPC-TE)

The hydroxyl value (OHV) of PPCDL was obtained by titration using an acetic anhydride–methylimidazole–dimethylformamide acylation method, averaging five parallel measurements. The moisture content of PPCDL was measured using Karl Fischer Coulometric Titration on a micro-water analyzer. Only PPCDL samples with moisture content < 100 ppm were used in the reactions.

**Figure 1:**
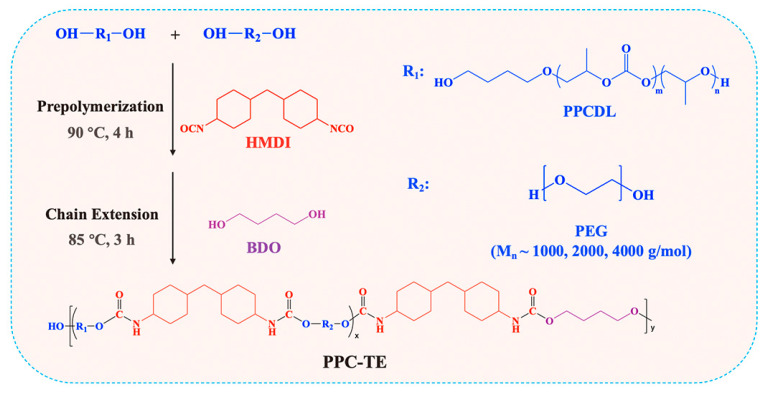
Schematic of PPC-TE synthesis.

The synthesis scheme of PPC-TE is illustrated in Figure 1. The specific formulations are detailed in Table 1. The syntheses were conducted in a three-necked round-bottom flask under nitrogen protection with mechanical stirring. The isocyanate-to-hydroxyl ratio (R = [NCO]/[OH]) was maintained at 1.15 with a fixed hard segment content of 25%.

General procedure (exemplified for PPC-TE2000-10): PPCDL (50.0 g, 22.1 mmol) and PEG2000 (5.56 g, 2.8 mmol) were added to the flask and vacuum dried at 110 °C for 3 h (a dual-stage rotary vane vacuum pump was used for vacuum treatment at a pressure of −0.1 MPa). After cooling to 60 °C, tin (II) 2-ethylhexanoate (0.23 g, 0.3 wt%), HMDI (17.8 g, 60.9 mmol) and DMF (25 mL) were added. The mixture was heated to 90 °C and prepolymerized for 4 h. After cooling to 60 °C, BDO (2.53 g, 28.1 mmol) and DMF (24 mL) were added. The chain extension reaction proceeded at 85 °C for 3 h. Finally, the product was precipitated out in ethanol and dried at 80 °C under vacuum for 48 h. The PPC-TE films were fabricated by preheating at 140 °C for 8 min, followed by hot pressing (8 min) and cold pressing (6 min). The PPC-TE samples were systematically labeled as PPC-TE-*x*-*y*, where *x*, *y* represent the molecular weight and weight percentage of the used PEG, respectively. A CO_2_-based thermoplastic polyurethane without the PEG segment (PPC-T) was synthesized for comparison.

### 2.3. Characterization Methods

FT-IR spectra were acquired on a Thermo Scientific Nicolet iS50 spectrometer (Thermo Fisher Scientific, Waltham, MA, USA) in ATR mode (400–4000 cm^−1^, resolution 1 cm^−1^). The molecular weight (*M*_n_) and dispersion index (PDI) were determined using a Shimadzu gel permeation chromatography (GPC) system. During testing, THF was used as the solvent (1.0 mL/min) and polystyrene (PDI = 1.02) was used as the standard sample.

The glass transition temperatures (*T*_g_s) were measured via differential scanning calorimetry (DSC-204, NETZSCH, Selb, Germany) at a heating rate of 10 °C/min under N_2_ flow. Thermal stability was assessed by thermogravimetric analysis (TGA, PerkinElmer TGS-2) from 30 to 600 °C at a heating rate of 10 °C/min under N_2_ flow.

The optical transmittances and hazes were measured in accordance with the standard specification (GB/T 2410-2008) using a WGT-S transmittance/haze tester (WGT-S, Shanghai Shenguang Instrument and Meter Co., Ltd., Shanghai, China). The thickness of the samples used for the transmittance test was 0.3 mm ± 0.08 mm.

Shrinkage was tested under the following procedure: the films were placed flat on 3.2 mm thick embossed glass, heated in an oven to 120 °C for 3 min, cooled to room temperature, and the length and width at the minimum distance were measured.

The thermomechanical properties were evaluated using a DMA 242D (NETZSCH., Germany) in a single cantilever mode. All tests were performed in the range of −180 °C to 190 °C at a heating rate of 2 °C/min and with an oscillation frequency of 1 Hz.

The tensile properties were determined according to GB/T 1040-92 using a universal testing machine (CMT 4204, Shenzhen SANS., Shenzhen, China) at 50 mm/min. The Shore A hardness was measured using an LX-A durometer (Guangzhou Sanliang Technology Co., Ltd., Guangzhou, China).

The water vapor permeability (WVP) was measured at 38 °C and 100% RH using a Permatran-W 3/61 (MOCON Inc., Minneapolis, MN, USA) according to ASTM F1249. Film thickness ≈ 0.3 mm. Oxygen permeability (OP) was measured at 23 °C and 45% RH using an oxygen permeability tester (Y210, Biaoji, China) according to GB/T 1038-2000. Film thickness ≈ 0.3 mm.

The adhesion to glass was tested according to GB/T 2790-1995. Samples (width 10 mm ± 0.5 mm) were laminated (glass/PPC-TE film/TPT back sheet) in a vacuum laminator at 140 °C. Peel force was measured at 100 mm/min.

### 2.4. Methods for Encapsulating Solar Cells Using PPC-TE Films

Two different sizes of purchased polycrystalline silicon solar cells (52 mm × 10 mm and 156 mm × 156 mm) were encapsulated using PPC-TE films. The encapsulation method was as follows: First, the PPC-TE film and TPT film were cut to the same area as the silicon wafer. They were then stacked in a five-layer structure: glass/PPC-TE/silicon wafer/PPC-TE/TPT. Next, the stacked photovoltaic module was placed in a vacuum hot press. The temperature of the vacuum hot press was set to 90 °C, and a vacuum was applied for 6 min to ensure complete adhesion between the PPC-TE film, the solar cell, the glass, and the back sheet. Finally, the temperature of the vacuum hot press was increased to 140 °C, and a vacuum treatment was performed for 6 min to obtain the photovoltaic module encapsulated with PPC-TE film.

### 2.5. Recyclability and Long-Term Stability of Encapsulated Cell

The recyclability evaluation of the encapsulated solar cells was conducted in a tube furnace (OTF-1200X-60, Hefei Kejing Materials Technology Co., Ltd., Hefei, China) under controlled thermal degradation conditions. Polycrystalline silicon solar cells (52 mm × 10 mm, 0.5 V, supplied by Nantong Shunfeng, Nantong, China) were encapsulated with two respective films (PPC-TE2000-15 and EVA film, thickness: 0.3 mm). Both encapsulated cells featured a standardized five-layer architecture: glass/encapsulant film/silicon wafer/encapsulant film/glass. The encapsulated cells underwent thermal treatment at 350 °C for 4 h under a continuous N_2_ flow to simulate end-of-life pyrolysis conditions.

The long-term stability of the encapsulated solar cells was evaluated under dual aging protocols: (1) natural weathering via outdoor exposure in Guangzhou’s subtropical climate (temperature range: 27–38 °C; relative humidity: 68–91%), and (2) accelerated aging in a controlled climate chamber (60 °C, 85% RH). Polycrystalline silicon solar cells (78 mm × 39 mm, 1.0 V, Jiangsu Sol New Energy Technology Co., Ltd., Suzhou, China) were encapsulated with PPC-TE2000-15 film (thickness ≈ 0.3 mm) employing industrial vacuum lamination technology, forming a five-layer architecture consisting of glass, PPC-TE, silicon wafer, PPC-TE, and TPT back sheet. Key electrical parameters, including open-circuit voltage (*V*_oc_) and short-circuit current (*I*_sc_), were systematically monitored every 6 h using a digital multimeter (VICTOR 99, VICTOR, Shenzhen, China), with initial values established as baseline references.

## 3. Results and Discussion

### 3.1. Chemical Structure (FT-IR)

**Figure 2:**
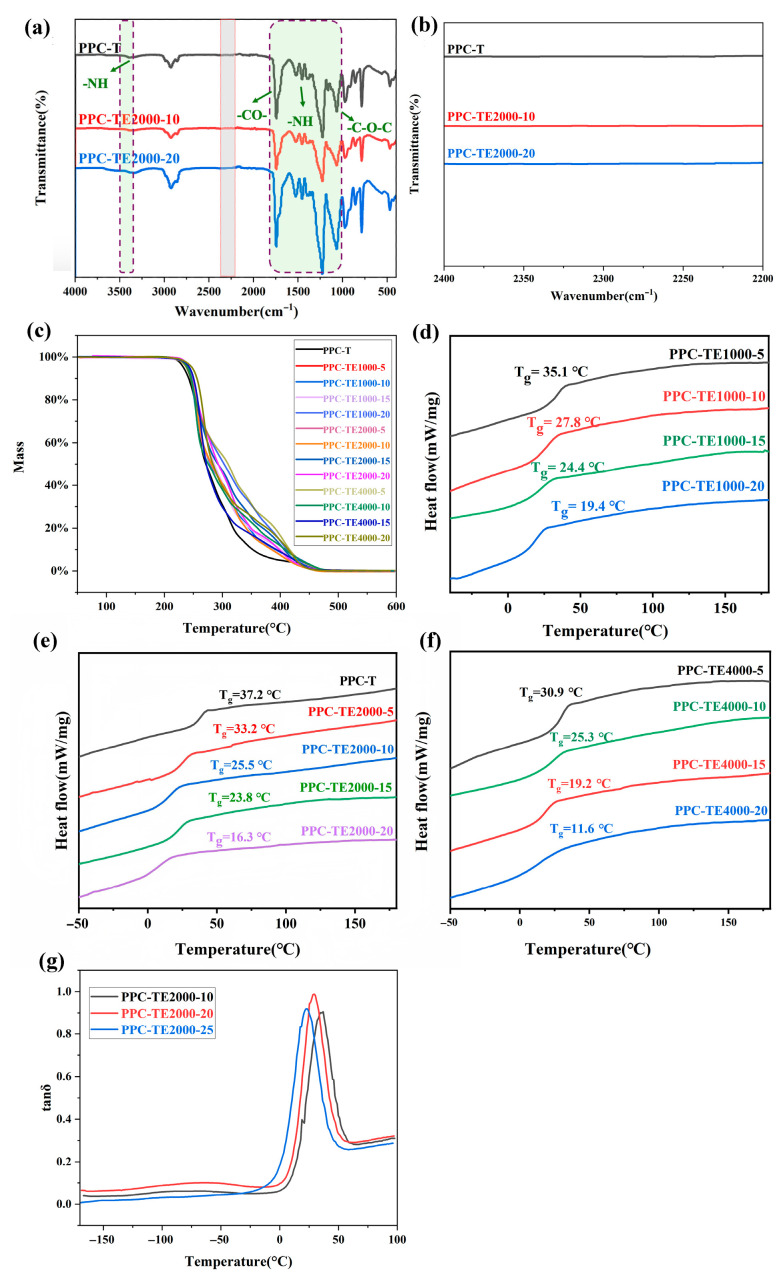
(**a**) FT-IR spectra, (**b**) enlarged view of FT-IR spectra, (**c**) TGA curves, (**d**–**f**) DSC curves and (**g**) evolution of loss factor (tan*δ*) of PPC-T and PPC-TEs.

FT-IR analysis (Figure 2a,b) confirmed successful PPC-TE synthesis via urethane bond formation. The absence of the characteristic isocyanate (-NCO) stretching frequency peak at 2270 cm^−1^ and hydroxyl group peak at 3460 cm^−1^ provides direct evidence of the complete addition reaction between isocyanate groups and hydroxyl groups. The absence of peaks at 1645–1636 cm^−1^ confirm no urea formation as a side reaction. The emergence of the urethane N-H stretch peak at 3290 cm^−1^, urethane carbonyl (C=O) stretch peak at 1700 cm^−1^, and other identifiable peaks including -N-H bend (1460 cm^−1^), C-O-C stretch (1080 cm^−1^), and C-O ester stretch (1240 cm^−1^) confirms the chemical structures of the synthesized CO_2_-based polyurethane (PPC-T and PPC-TEs).

### 3.2. Thermal Properties

The TGA results (Figure 2c) reveal a two-stage degradation profile for PPC-TEs, corresponding to the decomposition of hard segments and soft segments, respectively. The temperature at 5% mass loss (T_d5%_), indicative of thermal stability, exceeded 230 °C (range: 233–243 °C, Table 2), significantly higher than the maximum operating temperature (~85 °C) encountered by solar modules outdoors. Notably, T_d5%_ exhibits a slight increase with higher PEG content, suggesting enhanced thermal stability and broader usable temperature range. Crucially, all PPC-TE films undergo complete thermal degradation without residual carbon above 467 °C (Figure 2c), facilitating the potential eco-friendly recycling of silicon wafers and other device components. It represents a significant advantage over crosslinked EVA, as the high residual carbon content from the thermal decomposition of crosslinked EVA hinders wafer recovery.

The DSC analysis demonstrates effective modulation of the glass transition temperature (*T*_g_) through PEG incorporation (Figure 2d–f). Two key patterns are observed: (1) For a fixed PEG molecular weight (*M*_n_), *T*_g_ decreases progressively with increasing PEG content (e.g., PPC-TE2000: ~33.2 °C at 5 wt% PEG to ~16.3 °C at 20 wt% PEG, Table S1), attributed to the increased proportion of flexible PEG segments relative to the more rigid PPCDL; (2) At constant PEG content, *T*_g_ decreases with increasing PEG *M*_n_ (Table S2), likely due to the enhanced microphase separation and reduced crystallinity as the PEG chain length increased.

The DMA results confirm the DSC findings, showing a single tan*δ* peak shifting to lower temperatures with increasing PEG content (Figure 2g), confirming improved low-temperature flexibility.

### 3.3. Mechanical and Optical Properties

**Figure 3:**
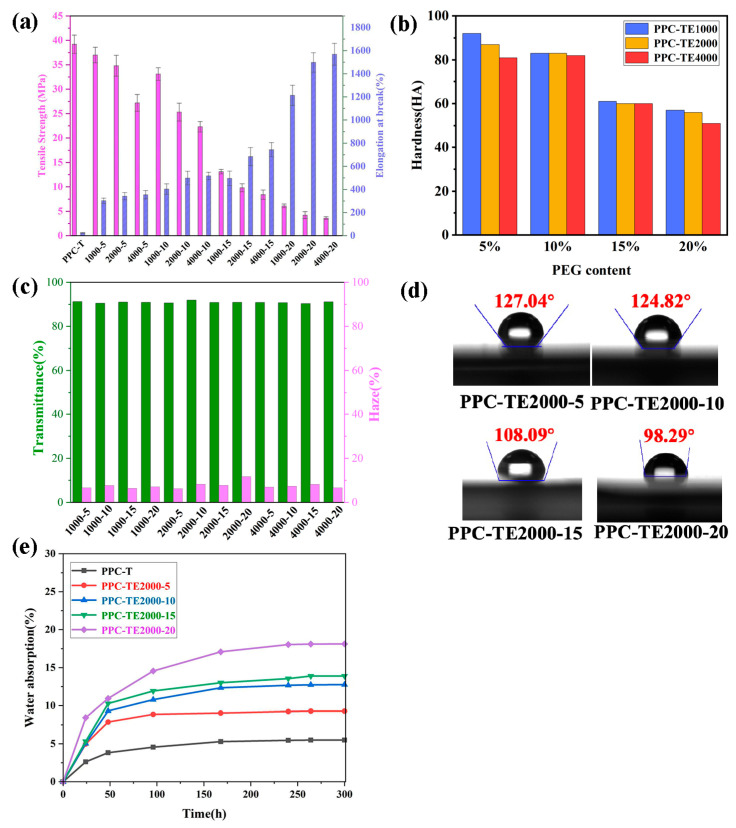
(**a**) Tensile strength and elongation at break, (**b**) hardness (shore A), (**c**) transmittance and haze of synthesized PPC-TE, (**d**) water contact angle (WCA) of PPC-TE2000, (**e**) water absorption of PPC-T and PPC-TE2000.

The mechanical adaptability of the PPC-TE films is readily tunable for solar cell encapsulation by varying the synthesis formulation of PPC-TE. Increasing the PEG content significantly enhances flexibility at the expense of strength (Figure 3a). The tensile strength increases dramatically from 37.0 MPa to 3.60 MPa (Table 3). Higher PEG molecular weight also improves elongation. This tunability allows films to be tailored for specific encapsulation requirements, balancing flexibility (need for adhesion, stress buffering, and impact resistance) with sufficient strength.

Shore A hardness measurements reflect similar trends (Figure 3b). The hardness decreases consistently with both increasing PEG content and molecular weight. The values range from 92 HA (PPC-TE2000-5 down to 51 HA (PPC-TE4000-20). Notably, PPC-TE films with 15 wt% PEG exhibit hardness values comparable to the crosslinked commercial EVA, suggesting similar handling characteristics during module assembly.

High optical transmittance and low haze are paramount for solar cell encapsulants to maximize light harvesting and minimize optical losses. Figure 3c demonstrates that varying PEG content (5–20 wt%) and molecular weight (1–4 kg/mol) have negligible detrimental effects on the optical clarity of the PPC-TE films. All samples exhibit transmittance exceeding 90% (up to 91.2%) and haze below 8.1%. This outstanding transparency, combined with tunability in other properties, makes the PPC-TE films highly suitable for solar cell encapsulation.

### 3.4. Surface and Barrier Properties

The hydrophobicity of the encapsulant surface is important for reducing water affinity. The water contact angle (WCA) measurements on PPC-TE2000 films (Figure 3d) show WCAs > 90°, confirming surface hydrophobicity. However, WCA decreases progressively with increasing PEG content due to the hydrophilic nature of PEG segments. The water absorption tests (Figure 3e) support this, showing significantly increased saturated water absorption (from ~4.7 wt% for PPC-TE2000-5 to ~12.6 wt% for PPC-TE2000-5) and faster absorption kinetics at higher PEG loading. These results highlight the need to balance PEG content for desired flexibility against maintaining sufficient hydrophobicity for long-term durability.

**Figure 4:**
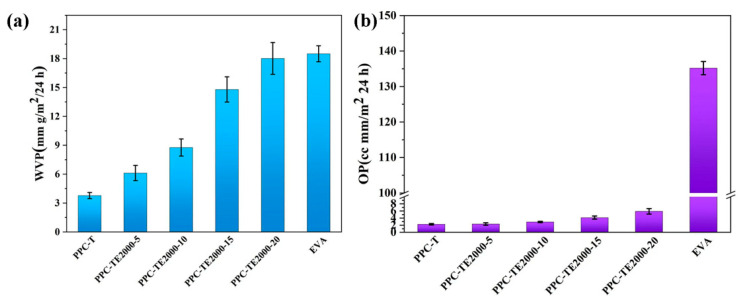
(**a**) WVP; (**b**) OP of synthesized PPC-T and PPC-TE2000.

The superior barrier performance against water vapor and oxygen is essential to prevent cell degradation. Figure 4a,b show that both OP and WVP increase with PEG content for PPC-TE2000 series, as PEG enhances the chain mobility and free volume of the polymers. Despite this increase, the PPC-TE films with PEG content below 15 wt% demonstrate significantly lower WVP and OP than the crosslinked commercial EVA (WVP: 18.5 g·mm·m^−2^·day^−1^; OP: 135.19 cc·mm·m^−2^·day^−1^). The specific data are provided in Table S3. For instance, PPC-TE2000-15 exhibits WVP of ~14.8 g·mm·m^−2^·day^−1^; OP of ~4.13 cc·mm·m^−2^·day^−1^. This enhanced barrier performance, particularly at the optimal PEG levels, is a key advantage of PPC-TE over the commercial EVA encapsulant.

### 3.5. Shrinkage Behaviors and Adhesive Properties

Low shrinkage during lamination ensures dimensional stability and tight component bonding, and avoids stress-induced cell damage. All of the PPC-TE films exhibit minimal shrinkage with transverse shrinkage < 3% and longitudinal shrinkage < 1.5 (Table 4), comparable to the commercial EVA films. This excellent dimensional stability, attributed to the thermal stability of PPC-TE, facilitates reliable module manufacturing and long-term performance.

**Figure 5:**
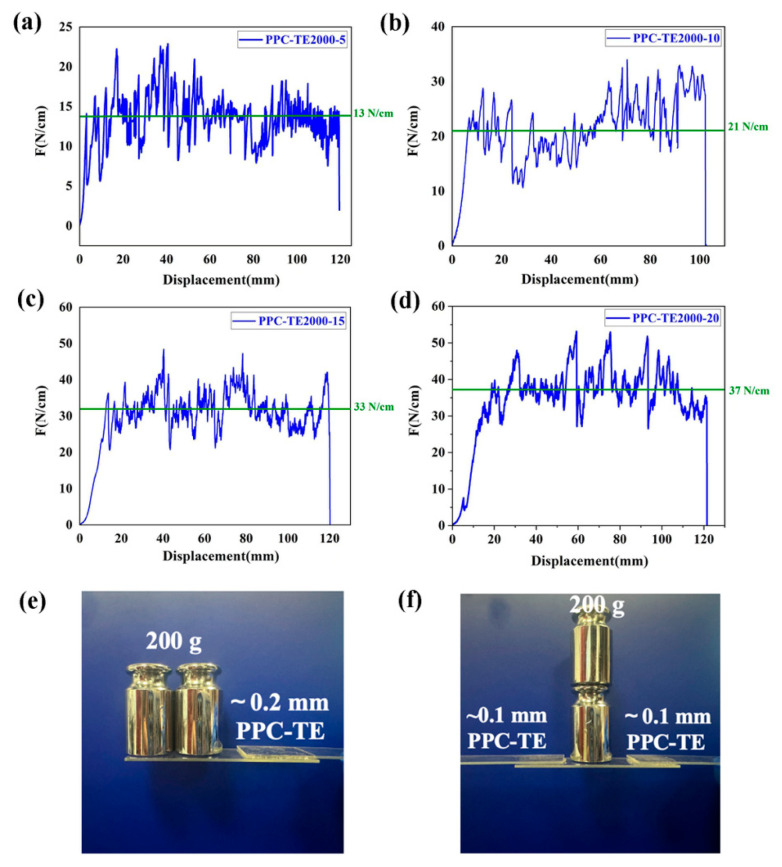
(**a**–**d**) Peel strength of PPC-TE2000; (**e**,**f**) photo of PPC-TE2000-15 support weights adhered to glass.

The strong adhesion to glass substrates is crucial for module integrity and longevity. The peel strength tests demonstrate excellent bonding of PPC-TE films to glass (Figure 5a–d). The adhesion strength increases with PEG content, reaching 37 N/cm for PPC-TE-2000-20. Moreover, after the peel test, the PPC-TE layer was completely detached from the glass substrate but remained adhered to the back sheet. A photograph of the tested sample on the back sheet is shown in Figure S1b, indicating that the PPC-TE itself remained intact without obvious signs of cohesive damage or material rupture. We preliminarily conclude that the failure mode is adhesive failure. A dramatic demonstration for this adhesion and cohesive strength is shown in Figure 5e,f. Here, a small piece of PPC-TE2000-15 film (0.1 mm thick, 2 × 2 cm^2^ area) bonded between glass slides successfully supports a 200 g weight equivalent to 1000 times its own weight, indicating exceptional potential for robust encapsulation (the measurement results for EVA under the same conditions are shown in Figure S1a).

### 3.6. Thermal Recyclability of Encapsulated Solar Cells

Currently, ethylene–vinyl acetate copolymer (EVA) is the most widely used solar cell encapsulant. However, its chemical stability after crosslinking poses significant challenges for recycling retired modules. The strong interfacial adhesion between EVA and glass, back sheet, and solar cells leads to silicon wafer breakage and silver grid damage during mechanical separation, reducing material recovery rate and economic value. Although pyrolysis can degrade EVA, it requires temperatures exceeding 500 °C, consumes substantial energy, and releases corrosive acetic acid and gases that contaminate metal components.

**Figure 6:**
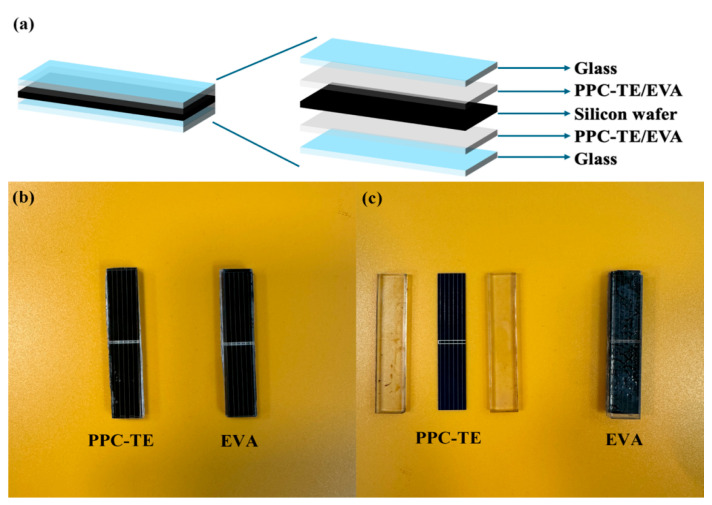
(**a**) Schematic of encapsulated solar cell structure; (**b**) visual comparison of cells encapsulated with PPC-TE (**left**) and EVA (**right**) films after lamination; (**c**) post thermal treatment at 350 °C for 4 h under N_2_ atmosphere.

To evaluate recyclability, solar cells (52 mm × 10 mm, 0.5 V) were encapsulated using PPC-TE2000-15 and EVA films (0.3 mm thickness) with a five-layer architecture (Figure 6a). As observed from Figure 6b,c, after thermal treatment, the PPC-TE film completely degraded. Due to the loss of the bonding effect of the PPC-TE interlayer, the original five-layer stacked structure (glass/PPC-TE/crystalline silicon wafer/PPC-TE/TPT) became two completely separable glass layers and an intermediate crystalline silicon wafer. After thermal treatment, PPC-TE completely decomposed. As a polycarbonate polyurethane, the primary decomposition products of PPC-TE are volatile gaseous products, such as carbon dioxide, water vapor, and nitrogen oxides. No solid residues were observed on the solar cell module. The complete decomposition of PPC-TE was also demonstrated by the mass change of the module before and after thermal treatment. The initial mass of the encapsulated module was 5.4685 g (including 0.1649 g PPC-TE), and the final mass of the collected glass and silicon pieces after treatment was 5.3034 g. Both glass layers are 52 mm × 10 mm × 3.2 mm in size. The solar cell is a commercially available polycrystalline silicon solar cell with dimensions of 52 mm × 10 mm. As shown in Figure 6c, the PPC-TE interlayer undergoes complete degradation after thermal treatment at 350 °C, which is also confirmed by the corresponding mass change. This degradation enables the clean separation of the glass and silicon wafer, allowing both components to be recovered and potentially reused. Therefore, it can be stated that the main components of solar cell modules encapsulated with PPC-TE are recyclable. This demonstrates PPC-TE’s superiority in enabling eco-friendly module recycling.

### 3.7. Long-Term Operation Stability of Encapsulated Solar Cells

**Figure 7:**
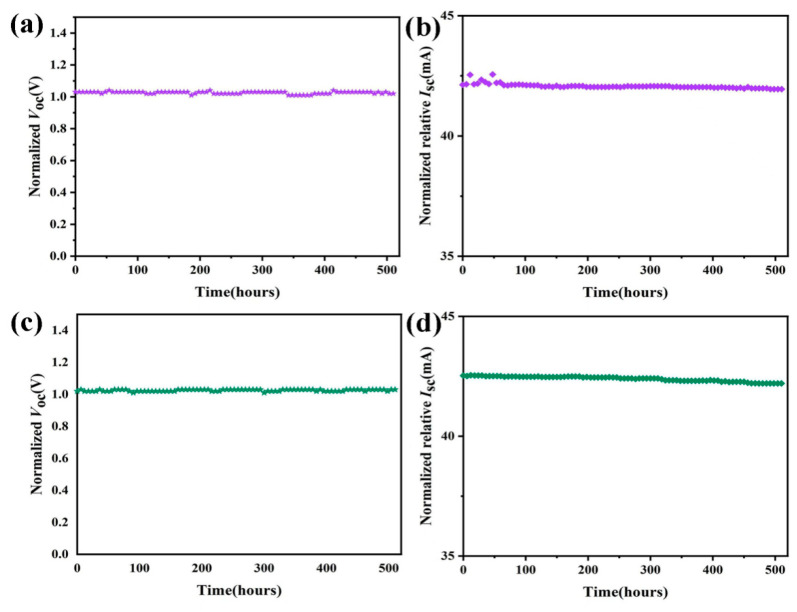
(**a**,**b**) Open-circuit voltage (*V*_oc_) and short-circuit current (*I*_sc_) measurements for solar cells in outdoor conditions; (**c**,**d**) *V*_oc_ and *I*_sc_ measurements in a constant temperature and humidity chamber (60 °C, 85% RH).

To evaluate the long-term stability of encapsulated photovoltaic devices, solar cells with a five-layer configuration using PPC-TE or EVA as an encapsulant were studied (Figure S1a,b). The experimental data, comprehensively illustrated in Figure 7, reveal exceptional stability across both test environments. Throughout the testing periods, *V*_oc_ and *I*_sc_ values maintained remarkable consistency with minimal deviation, while *I*_sc_ exhibited only marginal attenuation. To more directly assess the long-term stability of PPC-TE film-encapsulated solar cells in high-temperature, high-humidity environments, we connected four PPC-TE2000-15 film-encapsulated solar cells in series (Figure S2a) and subjected the resulting device to accelerated aging in a controlled climate chamber (60 °C, 85% RH) for 500 h. After 500 h, the device was removed and connected to a small LED bulb, which displayed normal light (Figure S4c). This persistent stability under varying environmental stresses substantiates the exceptional durability of the PPC-TE-encapsulated solar cells and validates their suitability for prolonged outdoor deployment in photovoltaic applications. The negligible performance degradation observed, particularly under high-humidity and elevated-temperature conditions, demonstrates the material’s robust protective capabilities, fulfilling critical requirements for commercial photovoltaic module encapsulation.

## 4. Conclusions

Currently, the mainstream encapsulants on the market, such as EVA and POE, struggle to simultaneously achieve both high barrier properties and strong adhesion. Moreover, the high crosslinking density of EVA makes it difficult to recycle and reuse encapsulated solar cell modules. This not only increases the cost of photovoltaic systems, but also contributes to environmental pollution. This study successfully developed a series of residue-free, thermally degradable CO_2_-based thermoplastic polyurethane (PPC-TE) encapsulants through molecular design by incorporating poly(ethylene glycol) (PEG) as a co-soft segment alongside CO_2_-derived polycarbonate diol (PPCDL). We precisely tailored the material properties through the systematic modulation of PEG content (5–20 wt%) and molecular weight (1000–4000 g mol^−1^). Through the autonomous and flexible regulation of PEG and PPCDL segments, PPC-TE can achieve a balance between high adhesion and high barrier properties. Moreover, the thermoplastic nature of PPC-TE enables it to be processed with ease.

The PPC-TE films exhibit a broad range of glass transition temperatures (T_g_ from 35.1 °C to 11.6 °C), mechanical properties (elongation at break: 302–1568%, hardness from 92–51 Shore A), and exceptional optical clarity (transmittance > 90%, haze < 8.1%). The films exhibit superior barrier properties (with WVP as low as 14.8 g mm m^−2^ day^−1^ and OP reduced to 4.13 cc mm m^−2^ day^−1^) at optimal PEG loading. These films exhibit robust interfacial adhesion (peel strengths up to 37 N cm^−1^), exceptional load-bearing capacity (supporting 1000× self-weight), and dimensional stability (<3% shrinkage). Their fully thermal degradation above 350 °C facilitates the eco-friendly end-of-life recycling of silicon wafer and module components.

From the perspective of industrial applications, the PPC-TE material system exhibits the following potential advantages: (1) Raw materials are readily available, and the synthesis process is simple. The core raw materials of PPC-TE (diols and isocyanates, etc.) have an industrial supply base. Its synthesis process is relatively simple, without complicated steps or harsh conditions. This is conducive to the stable control of the production process and large-scale scaling. (2) There is potential for cost-effectiveness throughout the entire life cycle. The biggest potential cost advantage of PPC-TE materials lies in its thermally decomposable and recyclable characteristics. This characteristic allows the glass, cells, and encapsulation materials to be effectively separated and recycled through heat treatment when the encapsulated components are decommissioned. From the perspective of the entire life cycle, this not only reduces the environmental burden, but also provides a long-term economic competitive advantage. This study explores the potential of novel thermoplastic polyurethane (PPC-TE) as a photovoltaic encapsulation material. However, we are also keenly aware that the mainstream material in the current photovoltaic encapsulation industry is still crosslinked EVA. Existing industrial equipment systems for lamination and transport are also largely built around the properties of EVA films. As a thermoplastic material, PPC-TE’s processing technology (such as temperature-pressure windows and cooling and shaping requirements) differs fundamentally from that of EVA, which has thermosetting crosslinking properties. This may pose challenges in process adaptation during the early stages of industrialization. Based on this, future research will focus on the synergistic optimization of material design and process compatibility. Specifically, a key direction is to explore the introduction of controllable crosslinking networks into the polyurethane molecular structure. Through molecular design, the material can be endowed with higher dimensional stability and water and oxygen barrier properties. More importantly, by controlling its rheological and curing behavior, the aim is to enable the new material to be maximally compatible with existing EVA lamination processes and equipment, thereby reducing equipment modification costs and process switching barriers during industrialization, paving the way for the new material to move from the laboratory to large-scale application. Recently, self-healing materials have demonstrated significant potential in the field of solar cell encapsulation, primarily through the use of dynamic reversible chemical bonds to achieve the autonomous repair of microscopic damage in the encapsulation layer. This technology can markedly enhance the long-term reliability of components under harsh environmental conditions such as humidity, heat, ultraviolet radiation, and mechanical stress. It is particularly suitable for emerging applications that are highly sensitive to stability, such as perovskite solar cells and flexible photovoltaics. If future efforts can overcome challenges related to cost and process compatibility, and integrate intelligent responsiveness with recyclable design, this approach holds promise as a key technological breakthrough for achieving ultra-long component lifespan and sustainable full-life-cycle development.

In summary, PPC-TE films represent a significant advancement in sustainable, high-performance PV encapsulation materials. Their tunable properties, fully thermal degradability, and overall performance make them suitable for large-scale production and application.

## Figures and Tables

**Table 1:** Synthesis parameters and molecular weights of PPC-TEs.

Sample	PEG M_n_ ^a^ (g/mol)	PEG Content (wt%)	M_n_ ^b^ (kDa)	PDI
PPC-T	-	0	51.5	1.87
PPC-TE1000-5	1000	5	68.2	1.88
PPC-TE1000-10	1000	10	56.3	1.82
PPC-TE1000-15	1000	15	54.2	1.85
PPC-TE1000-20	1000	20	49.3	1.73
PPC-TE2000-5	2000	5	78.5	1.84
PPC-TE2000-10	2000	10	89.7	1.85
PPC-TE2000-15	2000	15	82.7	1.74
PPC-TE2000-20	2000	20	80.5	1.66
PPC-TE4000-5	4000	5	89.5	2.01
PPC-TE4000-10	4000	10	78.2	1.96
PPC-TE4000-15	4000	15	83.5	2.03
PPC-TE4000-20	4000	20	87.6	1.96

^a^. Determined by titration method. ^b^. Determined by GPC with THF as eluent and calibrated by polystyrene standard.

**Table 2:** T_d5%_ and T_d10%_ (°C) of all synthesized PPC-T and PPC-TEs.

Sample	PEG Content	T_d5%_ (°C)	T_max_ (°C)	Char Yield (%)
PPC-T	0 wt%	233.1	465.9	0
PPC-TE1000	5 wt%	233.6	452.4	0
	10 wt%	235.7	463.5	0
	15 wt%	239.2	465.1	0
	20 wt%	241.4	467.0	0
PPC-TE2000	5 wt%	236.8	452.4	0
	10 wt%	240.8	449.1	0
	15 wt%	241.1	459.8	0
	20 wt%	242.9	456.8	0
PPC-TE4000	5 wt%	237.9	466.1	0
	10 wt%	240.6	468.8	0
	15 wt%	243.5	451.8	0
	20 wt%	247.9	449.8	0

**Table 3:** Tensile strength and elongation at break of prepared PPC-T and PPC-TEs.

Sample	Tensile Strength (MPa)	Elongation at Break (%)
PPC-T	39.2 ± 1.87	24.0 ± 3
PPC-TE1000-5	37.0 ± 1.59	302 ± 22
PPC-TE1000-10	34.8 ± 2.14	341 ± 31
PPC-TE1000-15	27.2 ± 1.72	354 ± 36
PPC-TE1000-20	33.1 ± 1.30	403 ± 45
PPC-TE2000-5	25.3 ± 1.83	498 ± 59
PPC-TE2000-10	22.3 ± 1.07	517 ± 31
PPC-TE2000-15	13.1 ± 0.47	495 ± 63
PPC-TE2000-20	9.80 ± 0.82	684 ± 78
PPC-TE4000-5	8.40 ± 0.95	743 ± 61
PPC-TE4000-10	6.10 ± 0.39	1213 ± 87
PPC-TE4000-15	4.20 ± 0.71	1498 ± 84
PPC-TE4000-20	3.60 ± 0.28	1568 ± 94

**Table 4:** Shrinkage of PPC-T and PPC-TEs.

Sample	PEG Content	MD (%)	TD (%)
PPC-T	0 wt%	1.90 ± 0.23	0.79 ± 0.21
PPC-TE1000	5 wt%	1.65 ± 0.16	0.88 ± 0.12
	10 wt%	1.49 ± 0.15	0.72 ± 0.08
	15 wt%	1.85 ± 0.21	1.31 ± 0.19
	20 wt%	2.27 ± 0.25	1.33 ± 0.21
PPC-TE2000	5 wt%	1.77 ± 0.25	1.42 ± 0.23
	10 wt%	1.61 ± 0.19	1.24 ± 0.21
	15 wt%	2.01 ± 0.23	1.31 ± 0.15
	20 wt%	2.82 ± 0.33	1.18 ± 0.19
PPC-TE4000	5 wt%	1.34 ± 0.14	0.65 ± 0.07
	10 wt%	1.73 ± 0.28	0.73 ± 0.13
	15 wt%	2.07 ± 0.31	1.21 ± 0.21
	20 wt%	2.00 ± 0.29	1.17 ± 0.18

## Data Availability

The original contributions presented in this study are included in the article/Supplementary Materials. Further inquiries can be directed to the corresponding authors.

## Supplementary Materials

The following supporting information can be downloaded at: https://www.mdpi.com/article/10.3390/nano16090503/s1, Figure S1: (a) Peel Strength of EVA, (b) failure mode photograph of PPC-TE; Figure S2: Photo of EVA support weights adhered to glass; Figure S3: (a) Schematic diagram of the structure of a polycrystalline silicon solar cell after packaging; (b) Photo of a solar cell encapsulated with PPC-TE2000-15 film; Figure S4: (a) Photo of four solar cells encapsulated with PPC-TE2000-15 films connected in series; (b) Brightness of LED bulb before accelerated aging; (c) Brightness of the LED bulb after 500 h of accelerated aging of the series-connected solar cells in a controlled climate chamber (60 °C, 85% RH); Table S1: the exact onset values of all synthesized PPC-T and PPC-TEs; Table S2: *T*_g_ (°C) of all synthesized PPC-TE; Table S3: WVP and OP of PPC-T, PPC-TE2000 and EVA.

## Abbreviations

The following abbreviations are used in this manuscript:

BDO	1,4-Butanediol
DMF	N, N-dimethylformamide
EVA	Ethylene-vinyl acetate
EtOH	Ethanol
HMDI	Dicyclohexylmethane 4,4′-diisocyanate
OP	Oxygen permeability
PEG	Polyether glycol
PPCDL	Polycarbonate diol
PSC	Perovskite solar cells
PVB	Polyvinyl butyral
POE	Polyolefin elastomer
TPU	Thermoplastic polyurethane
*T*_g_	Glass transition temperature
UV	Ultraviolet
WCA	Water contact angle
WVP	Water vapor permeability
