# Supplementary material for: Fully Thermally Decomposable CO2-Based Thermoplastic Polyurethane Encapsulation Films for Photovoltaic Cells: Mechanical, Barrier and Recycling Aspects
Source: Nanomaterials (Basel). 2026 Apr 22;16(9):503. doi: 10.3390/nano16090503 (PMC13164919; doi:10.3390/nano16090503)
Supplement: Supplementary file 1 [file nanomaterials-16-00503-s001.zip › nanomaterials-4250589-supplementary.pdf]

# Fully thermally decomposable $CO_2$-based thermoplastic polyurethane encapsulation films for photovoltaic cell : mechanical, barrier and recycling aspects

**Yuting Ouyang [1], Jizhi Ai [1], Min Xiao [1], Dongmei Han [2], Sheng Huang [1], Shuanjin Wang [1,*] and Yuezhong Meng [1,2,3,*]**

[1] The Key Laboratory of Low-Carbon Chemistry & Energy Conservation of Guangdong Province/State Key Laboratory of Optoelectronic Materials and Technologies, School of Materials Science and Engineering, Sun Yat-sen University, Guangzhou 510275, China; ouyyt7@mail2.sysu.edu.cn (Y.O.); aijzh@mail2.sysu.edu.cn (J.A.); stsxm@mail.sysu.edu.cn (M.X.); huangsh47@mail.sysu.edu.cn (S.H.)

[2] School of Chemical Engineering and Technology, Sun Yat-sen University, Guangzhou 510275, China; handongm@mail.sysu.edu.cn

[3] Institute of Chemistry, Henan Academy of Sciences, Zhengzhou 450000, China

* Correspondence: wangshj@mail.sysu.edu.cn (S.W.); mengyzh@mail.sysu.edu.cn (Y.M.)

**Table S1. the exact onset values of all synthesized PPC-T and PPC-TEs**

| Sample | PEG content | $T_{d5\%}$ (°C) |
|---|---|---|
| **PPC-T** | **0 wt%** | 42.7 |
| **PPC-TE1000** | **5 wt%** | 42.4 |
| | **10 wt%** | 35.2 |
| | **15 wt%** | 31.8 |
| | **20 wt%** | 26.6 |
| **PPC-TE2000** | **5 wt%** | 38.3 |
| | **10 wt%** | 34.5 |
| | **15 wt%** | 30.3 |
| | **20 wt%** | 25.2 |
| **PPC-TE4000** | **5 wt%** | 37.9 |
| | **10 wt%** | 34.6 |
| | **15 wt%** | 25.7 |
| | **20 wt%** | 25.1 |

**Table S2. $T_g$ (°C) of all synthesized PPC-TE**

| Sample | PEG content | $T_g$ (°C) |
|---|---|---|
| **PPC-T** | **0 wt%** | 37.2 |
| **PPC-TE1000** | **5 wt%** | 35.1 |
| | **10 wt%** | 27.8 |
| | **15 wt%** | 24.4 |
| | **20 wt%** | 19.4 |
| **PPC-TE2000** | **5 wt%** | 33.2 |
| | **10 wt%** | 25.5 |
| | **15 wt%** | 23.8 |
| | **20 wt%** | 16.3 |
| **PPC-TE4000** | **5 wt%** | 30.9 |
| | **10 wt%** | 25.3 |
| | **15 wt%** | 19.2 |
| | **20 wt%** | 11.6 |

**Table S3. WVP and OP of PPC-T, PPC-TE2000 and EVA**

| Sample | PEG content | WVP ($g \cdot mm \cdot m^{-2} \cdot day^{-1}$) | OP ($cc \cdot mm \cdot m^{-2} \cdot day^{-1}$) |
|---|---|---|---|
| **PPC-T** | **0 wt%** | 3.78±0.32 | 2.25±0.21 |
| **PPC-TE2000** | **5 wt%** | 6.12±0.79 | 2.33±0.34 |
| | **10 wt%** | 8.77±0.89 | 2.90±0.17 |
| | **15 wt%** | 14.80±1.31 | 4.13±0.44 |
| | **20 wt%** | 18.02±1.65 | 5.91±0.77 |
| **EVA** | / | 18.50±0.83 | 135.19±1.87 |

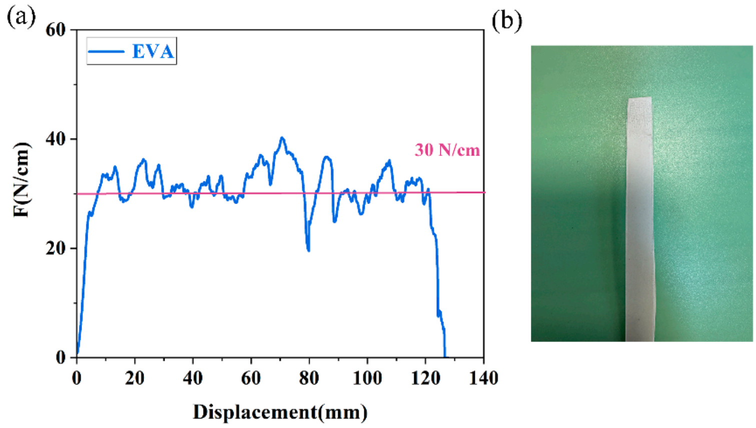


**Figure S1**. (a) Peel Strength of EVA, (b) failure mode photograph of PPC-TE

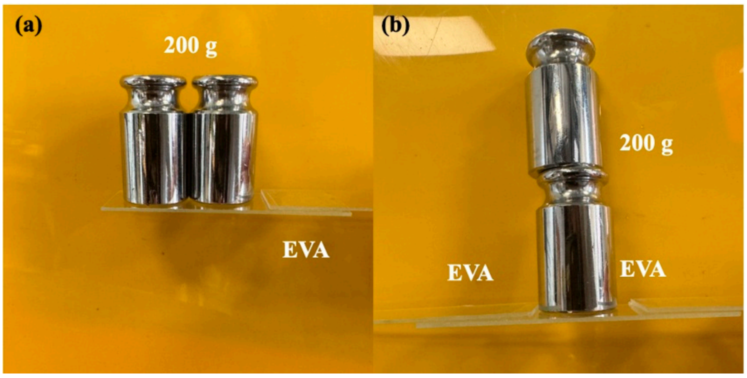


**Figure S2.** Photo of EVA support weights adhered to glass

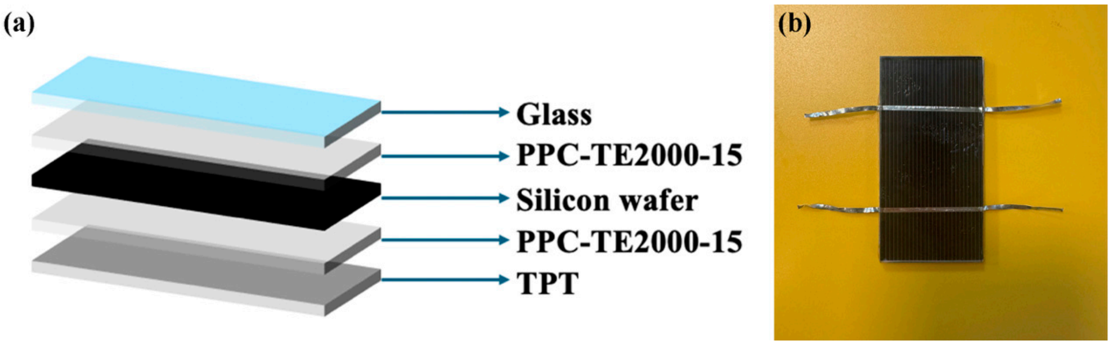


**Figure S3.** (a) Schematic diagram of the structure of a polycrystalline silicon solar cell after packaging; (b) Photo of a solar cell encapsulated with PPC-TE2000-15 film

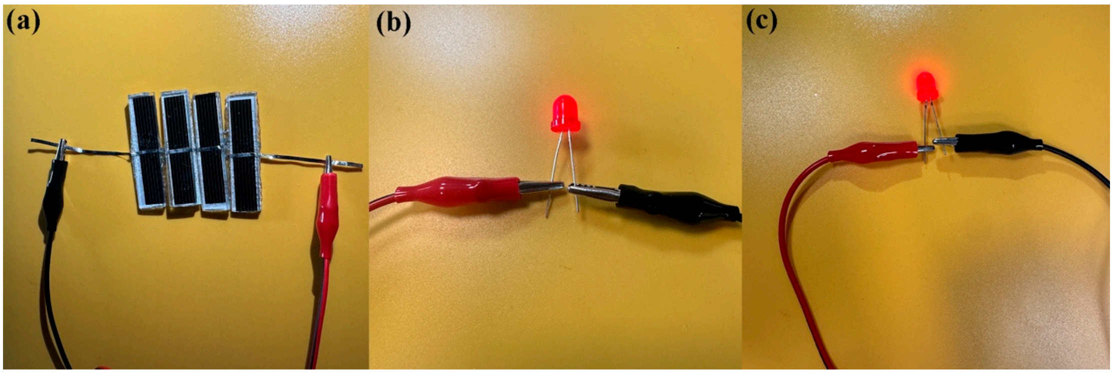

**Figure S4.** (a) Photo of four solar cells encapsulated with PPC-TE2000-15 films connected in series; (b) Brightness of LED bulb before accelerated aging; (c) Brightness of the LED bulb after 500 hours of accelerated aging of the series-connected solar cells in a controlled climate chamber (60°C, 85% RH)
